# An Approach Toward Automatic Classification of Tumor Histopathology of Non–Small Cell Lung Cancer Based on Radiomic Features

**DOI:** 10.18383/j.tom.2016.00244

**Published:** 2016-12

**Authors:** Ravindra Patil, Geetha Mahadevaiah, Andre Dekker

**Affiliations:** 1 Philips Research India, Bangalore, India and; 2 Department of Radiation Oncology (MAASTRO), GROW-School for Oncology and Developmental Biology, Maastricht University Medical Centre (MUMC), Maastricht University, Maastricht, The Netherlands

**Keywords:** radiomics, lung cancer, tumor histopathology, NSCLC

## Abstract

Non–small cell lung cancer contributes toward 85% of all lung cancer burden. Tumor histology (squamous cell carcinoma, large cell carcinoma, and adenocarcinoma and “not otherwise specified”) has prognostic significance, and it is therefore imperative to identify tumor histology for personalized medicine; however, biopsies are not always possible and carry significant risk of complications. Here, we have used Radiomics, which provides an exhaustive number of informative features, to aid in diagnosis and therapeutic outcome of tumor characteristics in a noninvasive manner. This study evaluated radiomic features of non–small cell lung cancer to identify tumor histopathology. We included 317 subjects and classified the underlying tumor histopathology into its 4 main subtypes. The performance of the current approach was determined to be 20% more accurate than that of an approach considering only the volumetric- and shape-based features.

## Introduction

Non–small cell lung cancer (NSCLC) accounts for 85% of all the lung cancers, and it is second most common cause of cancer in both men and women. According to the American Cancer Society, estimates for 2016 include an incidence of 224,390 new lung cancer cases in the USA (men, 117 920; women, 106 470) and 158,080 deaths due to lung cancer (men, 85 920; women, 72 160). Every year, more people die because of lung cancer than because of colon, breast, and prostate cancer combined. Further, 2 of 3 people diagnosed with lung cancer are aged ≥65 years, whereas <2% are aged <45 years (1). The different factors that can affect survival include genetic factors, clinical factors such as age and overall health, the size and grade of the tumor, and the histological subtype of NSCLC. With regard to the subtype, many studies have identified a link between the subtype and survival. For example, Ma et al. (2) recently showed that patients with adenocarcinoma have worse prognosis compared with those with nonadenocarcinoma subtypes. Similarly, Yano et al. (3) recently showed that squamous cell carcinoma has worse outcomes compared with other NSCLC subtypes, and concluded that surgical management should be different for different subtypes. Given this knowledge, it is imperative to classify NSCLS based on histology. Currently, biopsy is performed to identify the subtype, involving an invasive procedure and requiring multiple biopsies from the site of interest. Hence, it is painful, costly, and not without risk (4).

In recent years, noninvasive diagnosis and screening of lung cancer have been emphasized upon (5, 6). An advanced technique named “Radiomics” that involves the extraction of large quantitative features, resulting in conversion of images into higher-dimensional mineable data, can be used for building decision support systems. This is in contrast to the traditional practice of treating medical images as pictures used solely for visual interpretation. The output from a Radiomics analysis contains first-, second-, and higher-order statistical data derived from the entire image or a particular region of interest. It can be performed with tomographic images from computed tomography (CT), magnetic resonance imaging, and positron emission tomography studies. Aerts et al.'s seminal work (7) showed that radiomic features have prognostic power when used for patients with lung and head and neck cancer. In addition, several studies on NSCLC exist, for example, Al-Kadi et al., Cook et al., and Ganeshan et al. (8–10), in which texture analysis is applied to predict lung cancer outcomes on the basis of factors associated with tumor texture. In this study, we have hypothesized that radiomic texture features can noninvasively classify the different NSCLC subtypes.

The aim of this study is to perform a radiomic analysis to identify tumor histopathology in NSCLC and classify into squamous cell carcinoma, adenocarcinoma, large cell carcinoma, and not-otherwise-specified NSCLC. We extracted radiomic features and evaluated their ability to classify tumor histopathology and assessed how these features compare with nonradiomic “normal” features (eg, volume or diameter).

## Methodology

**Figure 1.**
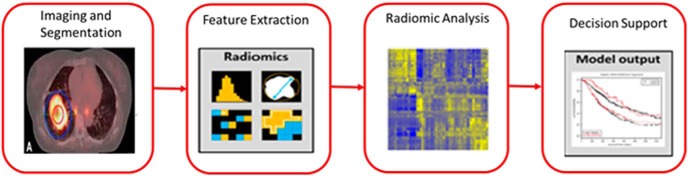
The Radiomics workflow. Images are taken partly from Aerts HJ et al. (7) with permission.

The applied methodology involves image acquisition, segmentation, feature extraction, and model validation to identify the histology of an underlying tumor. Figure 1 summarizes the aforementioned approach. The approach involved characterizing the tumor region by extracting radiomic features, which were earlier considered to be either redundant or inapplicable for clinical outcome. The obtained CT image is segmented to define the tumor region, and in this study, gross tumor volume is used. The segmented region is used to extract features based on tumor intensity, shape, and texture. The extracted features are analyzed to build a decision support system to identify tumor histopathology.

### Data

The data used in this study were obtained from The Cancer Imaging Archive (http://www.cancerimagingarchive.net/) from the collection of NSCLC-Radiomics. In all, 317 patients diagnosed with NSCLC were considered, of which, 40 patients were diagnosed with adenocarcinoma, 108 with large cell carcinoma, 110 with squamous cell carcinoma, and 59 with not otherwise specified. The demographic distribution is mentioned in Table 1. The ground truth of the tumor region was provided by a radiation oncologist in the DICOM-RTSTRUCT file; hence, no explicit segmentation algorithm was used for the tumor regions' identification. The data were neither preprocessed nor normalized so as not to devalue any clinical information present in the images.

**Table 1.** Distribution of Subject Characteristics

Subject Characteristics	Adenocarcinoma	Large Cell Carcinoma	Squamous Cell Carcinoma	Not Otherwise Specified
Number of Subjects	40	108	110	59
Male	20	65	70	41
Female	20	43	40	18
Mean Age (years)	67.2	66.9	70.2	65.6

### Feature Extraction

An important sub-step of Radiomics is the high throughput extraction of quantitative image features. In total, 431 imaging features were extracted from the tumor site identified by the manual delineation performed by the radiation oncologist. The feature-extraction algorithm was custom-implemented in Matlab (The MathWorks, Inc., Natick, Massachusetts). The quantitative imaging features were divided into 4 subgroups, namely, (1) first-order statistical features (2) shape- and size-based features, (3) textural features, and (4) multiscale wavelet, which describe tumor phenotypes. The first-order statistical features provide distribution of voxel intensities within the CT image via commonly used and basic metrics (eg, energy, entropy, and kurtosis). The shape- and size-based features provide tumor compactness, volume, and area metrics along with how spherical, rounded, or elongated the tumor is. The first-order statistical features represent only the information related to gray-level distribution and do not provide pertinent information regarding the relative position of various gray-level across images. Then, we computed textural features using gray-level co-occurrence and gray-level run-length texture matrices. All voxel intensities were resampled into equally spaced bins that were 25 Hounsfield units wide. The former step reduces image noise and normalizes the intensities, allowing direct comparison of all calculated textural features between patients. The matrices were determined considering 26 connected voxels in all 13 directions in 3 dimensions. The wavelets feature helps decouple original images into high and low frequencies. In this analysis, we applied one level undecimated 3-dimensional wavelet transform on the gross tumor volume. The original image is decomposed into 8 levels of wavelet decomposition (X_LLL_, X_LLH_, X_LHL_, X_LHH_, X_HLL_, X_HLH_, X_HHL_, and X_HHH_), where L and H denote low pass and high pass, respectively. For example, X_HLH_ is interpreted as a high-pass sub-band resulting from directional filtering in x with high pass, along y with low pass, and along z in high pass.

$$X_{LLH}(i,j,k)=\overset{N_{H}}{\underset{p=1}{\sum }}\overset{N_{L}}{\underset{q=1}{\sum }}\overset{N_{H}}{\underset{r=1}{\sum }}H(p)L(q)H(r)X(i+p,j+q,k+r)$$

Where *N_H_* is the length of filter H and *N_L_* is the length of filter L.

For each decomposition obtained above, we computed first-order statistical and textural features as mentioned earlier. The feature vector length for each subject was 431.

The first-order statistical, shape- and size-based, textural features, and wavelet decomposition features account for 14, 8, 33, and 376 features, respectively.

### Classification

Because data classes were skewed and to eliminate bias in learning due to unequal samples in each class, the SMOTE (Synthetic Minority Over-sampling Technique) algorithm (11) was applied on the data set. Two data models were built in this experiment, the first was the radiomic model that contained all the radiomic features extracted in the above step and the second was the normal model consisting of typical normal features, which were a subset of radiomic features consisting Energy, Entropy, Kurtosis, Maximum, Mean, Mean Absolute Deviation, Median, Minimum, Range, RMS Value, Skewness, Standard Deviation, Uniformity, Variance, Compactness, Maximum 3D diameter, Spherical Disproportion, Sphericity, Surface Area, Surface-to-Volume Ratio, and Volume). Following which, a multicategory support vector machine using R with package (e1071) was used for classifying the data into predefined classes. The results obtained were based on 10-fold cross validation on the data set. The experimental parameters set for the support vector machine were as follows: kernel type, rbf; gamma, 1.0; C, 0.1; epsilon: 10^∧^(−4). The aforementioned hyper parameters were selected on the basis of a grid search to yield the best accuracy value. Further, features were ranked on the basis of the built model to identify the most important predictive features that aided in the classification using the caret package in R.

## Results

**Figure 2.**
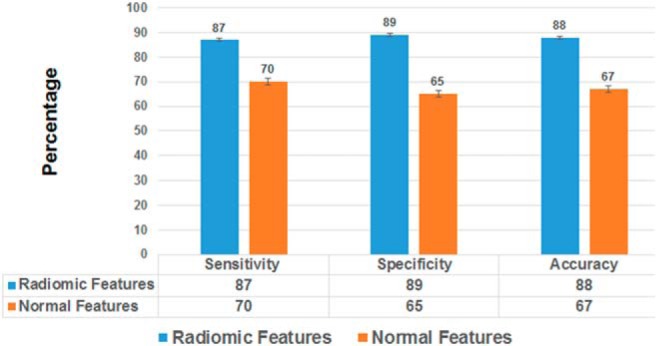
Classification metrics using radiomic and normal features.

The demographic of the data set included both male (60%) and female (40%) subjects, with their average age being 68 years. The metrics of histological classification obtained by considering radiomic features and those of normal features are shown in the Figure 2. It can be observed from Figure 2 that sensitivity in determining the histology using radiomic features is 87% and that using normal features is 70%. In addition, the specificity and accuracy considering radiomic features are 89% and 88%, respectively; however, those considering normal features are 65% and 67%, respectively. Further, the accuracy in identifying tumor histopathology using radiomic features showed 20% improvement (*P* < .0001) compared with that using normal features.

**Figure 3.**
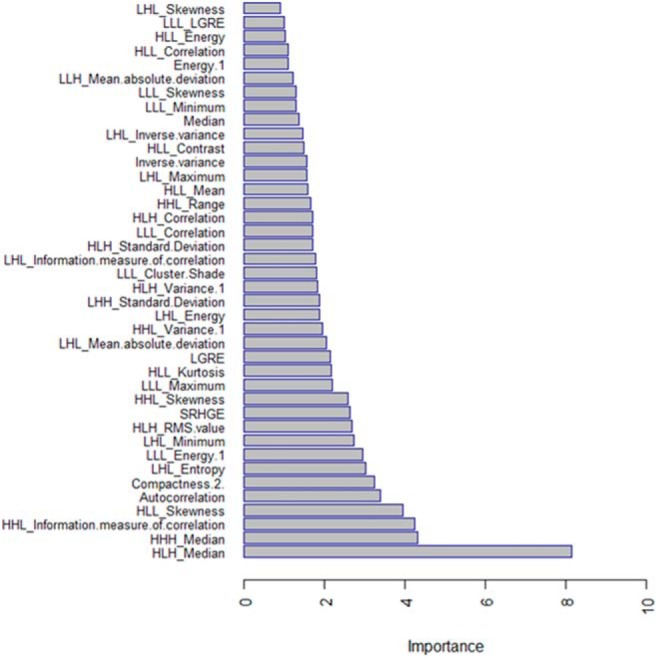
Radiomic features' ranking based on importance.

In addition, to rank the features based on importance value, feature ranking was used on the radiomic model. The top 40 radiomic features that aid in classification of tumor histopathology are shown in Figure 3. The top features contributing toward histological classification using radiomic features include HLH_Median, HHH_Median, HHL_Information_Measure_of_Correlation, HLL_Skewness, Autocorrelation, Compactness, LHL_Entropy, and LLL_Energy. It is interesting to note that volume as an independent feature does not rank as the top contributor for the classification and was ranked 53rd in the hierarchy. In addition, wavelet-based features dominate as top contributing features for the classification, strengthening the claim that additional features aid in better information extraction. Thus, in the classification of tumor histopathology, Radiomics can quantify phenotypical differences from medical images by using a large set of imaging features and provide more hidden information compared with normal features.

## Discussion

In this study, we adapted Radiomics to classify NSCLC, namely, adenocarcinoma, squamous cell carcinoma, large cell carcinoma, and not otherwise specified, by extracting imaging features from annotated tumor region. Limitations of this study include lack of validation of the external data set and the lack of noninvasive liquid biopsies (12), which could, in principle, provide a method to determine histological subtypes. The results of the study show that radiomic features have greater potential than normal features for classifying tumor histopathology. Our approach provides a noninvasive, fast, low-cost and repeatable method of investigating tumor histology, thus accelerating the development of personalized medicine. However, our method directly considered the tumor regions segmented by a radiologist; thus, future efforts should focus on integrating an automated segmentation algorithm in the workflow.
